# Feasibility of clinical newborn metabolic screening in a high-volume maternity center in Nepal

**DOI:** 10.1186/s44263-024-00040-x

**Published:** 2024-02-09

**Authors:** Janine Y. Khan, Kalpana U. Subedi, Shailendra B. Karmacharya, Prajwal Paudel, Dharma S. Manandhar, Rose Hennessy Garza, Keith A. Dookeran, Sunil R. Manandhar

**Affiliations:** 1 Department of Pediatrics, Ann & Robert H. Lurie Children’s Hospital, Chicago, IL USA; 2 Paropakar Maternity and Women’s Hospital, Thapathali, Kathmandu, Nepal; 3 Mother and Infant Research Activities (MIRA), Kathmandu, Nepal; 4 Joseph J. Zilber College of Public Health, University of Wisconsin-Milwaukee, Milwaukee, WI USA; 5 School of Public Health, Division of Epidemiology and Biostatistics, University of Illinois at Chicago, Chicago, IL USA; 6 The Cancer Foundation for Minority and Underserved Populations, Chicago, IL USA; 7 Kathmandu Medical College Teaching Hospital, Kathmandu, Nepal

**Keywords:** Newborn metabolic screening, Low–middle-income countries, Nepal

## Abstract

**Background:**

Strategic action plans around newborn health evaluation are needed, to address the high neonatal mortality rate in Nepal. Surveillance systems, like Newborn Metabolic Screening (NBS), could reveal unrecognized drivers of neonatal death. NBS is not routinely performed in Nepal. Our objective was to determine the feasibility of establishing NBS, and its acceptability among healthcare providers and parents, in Nepal.

**Methods:**

This prospective cohort study was conducted between November 2021 and May 2022 in term/late preterm infants born at Paropakar Maternity Hospital, Kathmandu, screening for 6 disorders that can be confirmed and managed locally. Staff were trained on dried-blood spot collection and transport protocols, performance metrics were established, and assays were performed at an accredited laboratory in Bangalore, India. Surveys were developed to determine acceptability among health-care providers and parents.

**Results:**

Of 835 parents approached for the study, 825 (98.8%) consented. Parental surveys showed that 92% considered “no cost” option most important in choosing to participate in the study. Samples were transported to laboratories in Kathmandu and Bangalore in 36 ± 24 h, and 4.75 ± 1 days, which exceeded expected metrics of 24 and 48 h, respectively. Results were communicated to parents by 9.5 ± 2 days, which was within the expected metric window of 14 days. Abnormalities were reported in 13 infants and included 5 hemoglobinopathy traits (4 Hb E and 1 Hb D), 3 congenital hypothyroidism, 2 glucose-6-phosphate dehydrogenase deficiency, 1 congenital adrenal hyperplasia, 1 elevated acylcarnitine, and 1 biotinidase deficiency. Healthcare providers surveyed (*n* = 116) showed that 67% reported a moderate understanding of NBS; all indicated that screening would be beneficial. Most cited early diagnosis and treatment, as well as, providing risk to future pregnancies as significant benefits. 90% thought screening should be routinely performed.

**Conclusions:**

We demonstrate that it is feasible to introduce NBS in Nepal. Transport metrics were longer than expected due to COVID pandemic travel restrictions; however, it was possible to deliver results to families within 2 weeks of birth. Parents overwhelmingly considered “no cost” option as the most important in choosing to screen. A government-sponsored program will be a key factor in establishing NBS in Nepal.

**Supplementary Information:**

The online version contains supplementary material available at 10.1186/s44263-024-00040-x.

## Background

Newborn screening (NBS) identifies newborns with serious treatable disorders to facilitate timely interventions that ameliorate adverse outcomes such as neurodevelopmental impairment, disability, and death. Universal NBS for Inborn Errors of Metabolism (IEMs) has played a significant role in public health strategies in the USA since 1966, by mitigating the burden of care in potentially curable disorders and making an impact on long-term health outcomes [1]. In Nepal, universal NBS is not available, however, a small number of NBS tests are sent to laboratories in India for analysis when IEMs are suspected, especially when infants are critically ill and/or dying, to determine the etiology of their illness, and NBS in these circumstances depends on the family’s ability to pay for testing.

Nepal, like many low and middle-income countries (LMIC), is seeking strategies towards achieving the United Nations Sustainable Development Goal (UNSDG) to reduce its current neonatal mortality rate (NMR) from 21/1000 to 12/1000 live births by 2030 [2, 3]. While childhood mortality has seen a 56% decline between 1973 and 2013 worldwide [4] the rate of reduction of neonatal death has been much slower and has in fact increased from 40% in 1990 to 47% in 2019 [5], and LMIC account for most of these deaths [6]. It is likely that the unknown causes of death in many cases of neonatal demise contribute to stagnant rates of NMR, and reducing the burden of death will be impeded if these causes remain undetermined.

Geographically, Nepal is divided into the flatland in the south, the hilly regions in the center, and the mountains in the north [7]. Racially, the Nepali population is divided into four major groups: Aryans, Mongoloids, Newars, and Tharus, and there are 142 castes and ethnicities reported in the 2021 Nepal census [8]. Within this diverse framework, coupled with limited healthcare options, the prevalence of metabolic disorders in Nepal remains largely unknown. The ethnically diverse population has appreciable consanguinity depending on ethnic and religious groups, some allow marriage with first cousins while others do not [9, 10]. In rural areas, the caste system is still adhered to and it has been reported that 21.6% of parents will not allow marriage outside their caste [11]. It is plausible that a proportion of neonatal deaths may be attributable to IEMs. NBS addresses an important gap in knowledge, not only in terms of establishing unexplained causes of neonatal death but also in cases of neurodevelopmental delay of unknown etiology. A retrospective study in neighboring India investigating known cases of IEMs in a pediatric intensive care unit showed that 36% had sequelae of irreversible intellectual disability [12]. A World Health Organization representative estimated that 1% of the Nepali population has severe neurological disability and 10–20% has milder mental health issues [13]. In fact, Nepal’s health profile shows a 1.63% prevalence of neurodevelopmental disabilities, of which 5.9% have intellectual disability [14]. Speculation exists that many undiagnosed cases of intellectual disability in Nepal may represent survivors of missed IEM diagnoses, for example, in Eastern Nepal, the rate of disability was estimated at 4.87%, of which 17% was reported to be due to an “inborn syndrome” (likely a combination of genetic and/or metabolic disorders) [15]. Early diagnosis is desirable because some IEMs respond to changes in diet or medication, for example, l-thyroxine for hypothyroidism. Early diagnosis and intervention for many of these potentially treatable disorders may make it possible to reduce neonatal morbidity and mortality.

We established a pilot study of NBS in term/late preterm infants born at the largest birthing hospital in Nepal, Paropakar Maternity Hospital, Kathmandu. The objectives of the study were to determine the feasibility of establishing NBS in Nepal, using a limited number of disorders that can currently be confirmed and managed in Nepal, utilizing a process map for sample collection, transport, analysis, and result communication to healthcare providers and families; and, to determine acceptability of introducing universal NBS among maternal-child health providers and, screened and enrolled parents, through Qualtrics surveys. Preliminary results of this work were presented as a scientific poster presentation at the 2022 American Academy of Pediatrics National Conference, Anaheim, California [16].

## Methods

This was a descriptive, prospective, cohort study. Training for participating physicians and nurses was conducted by two study personnel: SRM (Head of the Neonatal Intensive Care Unit [NICU] and Head of the Department of Pediatrics, Kathmandu Medical College Teaching Hospital), and KS (Medical Director of the NICU at Paropakar Maternity and Women’s Hospital); Paropakar Hospital’s Department of Pathology, and personnel from an Intermediate laboratory in Kathmandu. A hybrid model consisting of in-person and web-based NBS educational videos from PerkinElmer Inc. based on information in Clinical and Laboratory Standards Institute (CLSI) Standard NBS01 was adopted for this training session (https://www.youtube.com/watch?v=_UFAAF4cZ2o, https://www.youtube.com/watch?v=qg_h354MVaU&t=17s). Pre-packaged kits with equipment for Dried Blood Spot (DBS) collection, including gloves, lancet, alcohol prep, gauze pad, filter paper with form, and pouch, were provided by NeoGen Laboratory (Bangalore, India), an NBS laboratory accredited by the College of American Pathologists with established Kathmandu transport logistics. Training included DBS collection method (heel-stick sample of 0.5 mL blood), drying of DBS filter paper card on a dry, clean, flat, non-absorbent surface for a minimum of 4 h, filling out the attached form with patient address and phone number for communication tracking, labeling and identification of the sample, placement in a foil pouch, and storage in a cool dark closet prior to transport. Specimens were first taken to a laboratory in Kathmandu and then courier-shipped to NeoGen in Bangalore. The comprehensive fixed-cost service included the DBS pre-packaged kit, sample transfer to Kathmandu laboratory for packaging in batches and courier transport to Bangalore, sample analysis, communication of report to healthcare providers by email and web access, and repeat testing for equivocal results. Although all samples were assigned a unique identification number, authors involved in clinical care of the participating infants had access to patient identifiers during and after data collection, since it was important that they contact families with reports.

Metrics for feasibility were determined a priori by the study team, and included, sample collection competency, number of samples collected within the target range of 24–48 h after birth, time to delivery to Kathmandu laboratory (24 h), time to arrival at NeoGen (2 days), time between arrival at NeoGen and result communication to healthcare provider (72 h), time to result communication to family by providers (48 h), time between birth and result communication to family (14 days), number of families contacted with results (100%), and number of reports requiring confirmation/intervention (< 3%). Metrics data were obtained from actual time documented on the attached DBS form, and from NeoGen Laboratory reporting data.

Qualtrics surveys were developed for parents approached for the study and for healthcare providers. Surveys for parents were administered by written response immediately after the consenting process, and parents were asked to choose the response that best fits them, and respond “yes/no” to each item on the survey. Following a review of the literature highlighting common conditions reported during NBS in the region, specifically Nepal and India, we limited testing to conditions that could be medically managed in Nepal. Hence, NBS panels were determined as follows: *standard small panel* included testing for six disorders that could be confirmed and managed in Nepal: congenital hypothyroidism, congenital adrenal hyperplasia, glucose-6-phosphate dehydrogenase deficiency, galactosemia, hemoglobinopathy, and biotinidase deficiency; *high-risk expanded panel* (NeoGen panel with comprehensive testing of 72 disorders including conditions in the standard small panel, plus disorders of fatty acid oxidation, organic acid, amino acid, lysosomal storage, severe combined immunodeficiency and carnitine deficiency).

The study was conducted at Paropakar Maternity and Women’s Hospital, Kathmandu, Nepal, in partnership with Mother and Infant Research Activities (MIRA), during a 6-month period between November 2021 and May 2022. Samples were collected 24–48 h after birth, after obtaining written informed consent from a parent. Our aim was to obtain 800–850 DBS samples to show the reproducibility of collection, transport logistics, and result communication. Maternal and infant data were abstracted from admission records. Parents were approached during normal working hours, 8am to 4pm, Sunday to Friday, as Saturday is Nepal’s “weekend day”; and, using systemic sampling, every sixth admission to the post-natal nursery was included in the study. To minimize information bias, the data collection team was independent of the clinical care team.

**Fig. 1:**
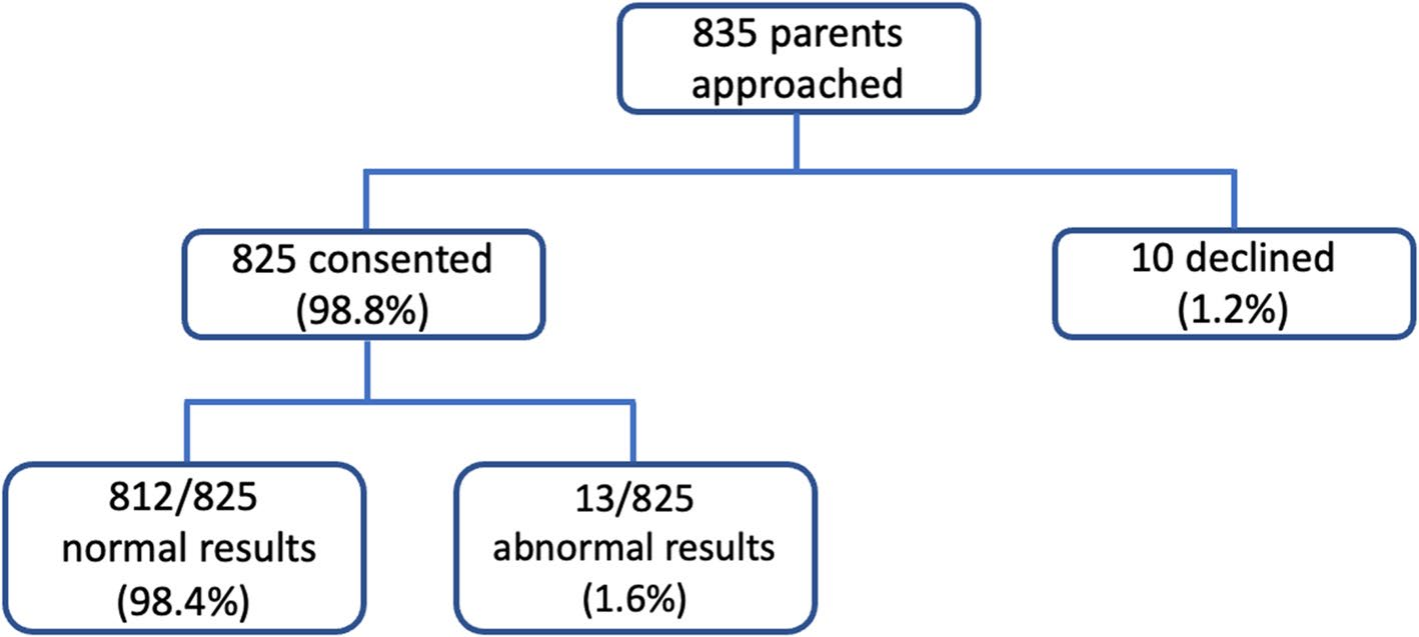
Flow diagram for study enrollment

Study inclusion criteria were infants born at greater than or equal to 34 weeks gestation and admitted to the post-natal nursery, who were tested with the *standard small panel*; infants at high-risk for IEM (i.e., unexplained death of a neonatal sibling, maternal history of repeated miscarriages, family history of neurodevelopmental impairment, family history of previously diagnosed IEM) who were admitted to the NICU and tested with the *high-risk expanded panel*; and any infant presenting to the NICU within the first 72 h of life with life-threatening sepsis and index of suspicion for IEM per physician discretion, who were also tested with the *high-risk expanded panel*. Study exclusion criteria were routine testing of preterm infants < 34 weeks gestation to avoid the complication of false positive results that may occur in preterm babies and require multiple repeat tests throughout hospitalization. However, physicians at Paropakar Maternity Hospital were allowed to send the high-risk expanded panel for infants with suspected IEMs in whom the level of illness was disproportionate to their diagnosis, and a few of these infants were < 34 weeks gestational age. Repeat testing was performed on all infants with abnormal results since it is not unusual for borderline abnormal results to normalize over time in unaffected infants, and confirmatory testing is required for all abnormal screening test results. Figure 1 shows a flow diagram for study enrollment. Ethical approval for the study was obtained from the Paropakar Maternity and Women’s Hospital Institutional Review Committee (Ref No. 6712014), Nepal Health Research Council (Ref No. 385), and Ann and Robert H. Lurie Children’s Hospital Institutional Review Board (IRB 2021–4591). Our study methodology conforms with all Strengthening the Reporting of Observational Studies in Epidemiology guidelines [17] (STROBE checklist—see Additional file 1).

## Results

Study enrollment is shown in the flow diagram (Fig. 1), and maternal and infant demographics are reported in Table 1. Median maternal age was 24 years. Most mothers (96%) were of lower middle/middle socioeconomic status [18], and almost three-quarters reported more than five prenatal visits including some prenatal visits in a hospital setting. Prenatal visits for most mothers were carried out at government health clinics and hospitals. These visits incur no cost to families, and the Nepali government provides cash incentives for delivery in government hospitals, of which Paropakar Maternity and Women’s Hospital is the largest in Nepal with 25,000 deliveries per year. The median gestational age was 39 weeks.

**Table 1:** Maternal and infant demographics

**Maternal**	
**Age (years)**	
Range	17–39
Median	24
**Socioeconomic status (SES)**	
Upper middle	0.5%
Lower middle/middle	96%
Lower	3.5%
**Antenatal visits**	
< 5	29%
> 5	71%
**Place of antenatal care**	
All or some hospital care	72%
Health posts only	26%
**Mode of delivery**	
Vaginal	80%
Cesarean section	20%
**Infant**	
**Gestation age (weeks)**	
Range	28–43
Median	39
**Birth weight (grams)**	
Mean ± SD	2985 ± 480
**Sex**	
Male	46%
Female	54%

Of 835 parents approached for the study, 825 parents (98.8%) consented. Most parents consenting to the study selected the following from a list of statements influencing their choice (Table 2): “I was clear about the best choice for my baby” and “I chose without pressure from others”, and almost all parents considered the “no cost” option most important in choosing to participate in the NBS study. Parents who declined to participate chose the following statements: “I don’t wish my child to be pricked or have blood taken” and “I don’t understand the purpose of newborn screening”.

**Table 2:** Parent survey—list of statements influencing choice for study participation

**Reasons for consent to NBS study participation**		
a	I would like to know that my child is healthy	24%
b	Screening is free	92%
c	Screening offers early detection and treatment of potential abnormal conditions	24%
d	The potential benefit to my child	12%
e	Other reason(s)	2%
**Reasons for choice to participate in NBS study**		
a	I knew the choices available to me	4%
b	I knew the benefits and risks of each choice	21%
c	I was clear about which choice was more important to me	21%
d	I had enough support from others to make a choice (others could be family, friends, healthcare provider)	31%
e	I chose without pressure from others	43%
f	I was clear about the best choice for my baby	43%
g	I felt sure about what to choose	10%
h	This decision was easy for me	24%
i	I am satisfied with my decision	18%
**Reasons for decline to NBS study participation**		
a	I prefer not to know if my child has a condition	0%
b	The results may not be accurate	0%
c	I do not wish my child to be pricked or have blood taken	50%
d	The thought of my child being experimented on	0%
e	I do not understand the purpose of newborn screening	40%
f	I do not think newborn screening is important	10%
g	Baby’s father or family member does not want baby to participate in the study	0%
h	Other reason(s).____	0%

Health provider metrics for acceptability (Table 3) showed that 92% of respondents were pediatricians with two-thirds having at least a moderate understanding of NBS and had sent a NBS test in the past. All agreed that NBS would be beneficial, with 90% indicating that NBS should be routinely performed in Nepal. The most common benefits cited were early diagnosis/treatment and providing an understanding of risk to future pregnancies. Hesitation regarding the implementation of NBS centered mainly around the unavailability of comprehensive management. Perceived challenges to NBS highlighted the cost of testing for families, unavailability of facility for analysis, delay in receiving results, and concerns regarding the ability to contact families with results.

**Table 3:** Health provider metrics for acceptability of newborn screening

**Health provider metrics (*n* = 114)**	
Have you sent a NBS test in the past? (yes)	72%
Understanding of NBS? (moderate)	67%
Would NBS be beneficial? (yes)	100%
Is the cost worth the benefit? (yes/no)	77%/5%
Should NBS be performed routinely in Nepal? (yes/no)	90%/3%
Likely to use NBS in the practice if available? (yes)	86%
**Benefits of NBS per healthcare providers (> one answer)**	
No benefit	0%
Early diagnosis	95%
Early treatment	89%
Education of parents	83%
Provide reason for unexplained death and/or neuro-disability	88%
Provide understanding of risk for future pregnancy	91%
Determine population prevalence of IEM	86%
**Hesitation regarding NBS implementation (> one answer)**	
Comprehensive management unavailable	72%
Conditions tested are not significant contributors to mortality/morbidity	22%
Diversion of funds from other areas of the healthcare system	36%
Social stigma	11%
No hesitation	17%
**Perceived challenges to NBS (> one answer)**	
Cost	100%
Availability of facilities for analysis	99%
Inadequate collection and processing	94%
Delay in receiving test result	93%
Time commitment for counseling and testing	94%
Ability to contact families to provide results	100%

Table 4 compares observed versus expected feasibility metrics. Sample collection competency was 99.9%. Time to arrival at NeoGen was longer than expected at 4.75 ± 1 day (mean ± SD), however the time between arrival at NeoGen and result communication to the provider was shorter than expected at 24 ± 8 h; therefore, the time between birth and result communication to families was 9.5 ± 2 days.

**Table 4:** Observed vs. expected metrics for feasibility of newborn screening

Metrics for feasibility	Expected outcome	Observed outcome
Sample collection competency	100%	99.9%
Sample collection within 24–48 h	100%	96.4%
Time to delivery to KTM intermediate lab (mean ± SD)	24 h	36 ± 24 h
Time to arrival at Bangalore lab (mean ± SD)	2 days	4.75 ± 1 days
Time to result communication to Provider after arrival in Bangalore Lab (Mean ± SD)	72 h	24 ± 8 h
Time to communication of result to family by healthcare provider (mean ± SD)	48 h	30 ± 18 h
Time between birth and result communication to family (Mean ± SD)	14 days	9.5 ± 2 days
Number of families contacted with results	100%	100%
Number of reports requiring confirmation/Intervention	< 3%	1.6%

Among the 825 infants participating in the study, 812 infants had normal results and 13 (1.6%) had abnormal reports on initial testing—five hemoglobinopathy traits (four Hb E and one Hb D), three congenital hypothyroidism, two G6PD deficiency, one CAH, one elevated acylcarnitine and one biotinidase deficiency. On repeat testing of infants with abnormal results the neonate with elevated acylcarnitine and one of the neonates with congenital hypothyroidism had normal results.

## Discussion

Our study is the first to demonstrate that it is feasible to build a program around NBS in Nepal, and with appropriate training, DBS samples were collected, stored, transported to Bangalore, India and analyzed efficiently. The previous pilot study in Nepal by Pandey et al. [19] is important as it demonstrates the need for NBS in Nepal to determine the prevalence of IEMs. However, unlike our clinical study, Pandey’s study was experimental with samples shipped to Switzerland in monthly batches, therefore results were not available for optimal management. Since many IEMs clinically present within 1–2 weeks of age, their methodology would not be effective for a NBS Program. Our study was conducted in real time with samples collected at 24–48 h of age as recommended by CLSI Standard NBS01, and samples were shipped 2–4 times per week to Bangalore, India to allow for a timely turnaround of results to healthcare providers and families, thus facilitating early confirmatory testing and intervention which is the main purpose of NBS.

The study team empirically set the target of 4–6 days as the turnaround time to healthcare providers, and 14 days as the upper boundary of turnaround time from birth to result communication to parents since we perceived that our greatest barrier in this pilot project would be our ability to contact families in a timely manner. In fact, the greatest challenge proved to be delayed transport to NeoGen Laboratory in Bangalore of 4–5 days instead of the anticipated 2-day window, due to a reduction in flights from Kathmandu resulting from Nepal’s COVID-mandated protocols. However, NeoGen was able to provide results to healthcare providers within 24 h of receiving samples, and providers were then able to contact the families between 12–48 h after receiving results. Without delays caused by alterations in transport within and outside Nepal during the COVID-19 pandemic, it is likely that almost all families would have received NBS results by 1 week after birth. This is relevant because one of the conditions tested on our *standard small panel* was congenital hypothyroidism. The true prevalence of congenital hypothyroidism (CH) in Nepal remains unknown. Mishra et al. estimated a prevalence of 4.5% (Confidence Interval 3.35–5.65) from relatively contemporary laboratory records between 2013 and 2020 [20] and showed that as many as two-thirds of cases were diagnosed between 3 and 12 months of age. The delay in diagnosis may be attributed to the fact that newborns tend to be asymptomatic at birth, presenting later in infancy with symptoms of hypotonia and/or delayed milestones indicative of irreversible neurodevelopmental impairment. 2020–2021 Consensus Guidelines [21] recommend initiation of replacement therapy as soon as possible after birth, optimally by 2 weeks of age, to avoid adverse neurocognitive outcomes [22]. Of all the conditions tested in our study, CH can be easily confirmed in Nepal and appropriately managed with levothyroxine therapy that is readily available and relatively inexpensive, for example, a 3-month supply may be as low as 250–300 Nepalese rupees (U.S. $1.80–$2.25). With an NBS Program, early diagnosis and treatment will reduce the CH-associated disease burden and optimize the health outcomes of affected neonates.

Despite our limited sample size, we were able to screen abnormalities in 1.6% of the study population. This is not representative of the true prevalence of IEMs because we tested for a limited panel of disorders. But we know that these diseases exist in Nepal because there are case reports and small, limited studies documented in the literature [19, 23]. The general lack of awareness of IEMs among Nepali pediatricians stems from the unavailability of an IEM screening facility. As a result, the diagnosis rate is low since only a small number of NBS tests are performed, usually in high-risk cases during their illnesses, due to limitations arising from economic factors, sample transport logistics, and perceived challenges with communication of results to families in remote areas. Additionally, the *high-risk expanded panel* is currently the only option for clinicians as the *standard small panel *was created specifically for our study and is not commercially available. Since Nepal does not have the capability and expertise to manage most IEMs in the *high-risk expanded panel*, physicians do not have the incentive to pursue a diagnosis. A fundamental change is needed to promote awareness of IEMs within the healthcare community and among policymakers in Nepal so that the current infrastructure is modified to allow for a seamless flow of knowledge, skills, and management of IEMs. We were able to raise awareness of the importance of NBS through a project dissemination initiative beginning in November 2022 through May 2023, aimed at sharing the results of our study with physician groups, Nepal Pediatric Society (NEPAS) and Perinatal Society of Nepal (PESON), as well as governmental and non-governmental agencies.

Our study shows it is entirely reasonable for Nepal to have NBS testing analyzed in India, and still achieve diagnosis and management in a timely manner, and NeoGen Laboratory has the capacity to perform this function for Nepal if universal testing is implemented. It is our opinion that Nepal is well-positioned for this type of development. The pediatric community appears overwhelmingly in support of an NBS program, as all providers surveyed considered that NBS would be beneficial, and the majority indicated that it should be performed routinely. They see value in NBS as a means of early diagnosis and treatment, helping to assess future pregnancy risk, and finding reasons for unexplained neonatal death and/or neuro-disability. However, healthcare providers’ enthusiasm for universal NBS is tempered by anticipated challenges, the foremost being the cost of testing that could not be met by many parents without governmental subsidy. The cost of NBS testing for our study was high by Nepali standard, at U.S.$21 (2791 Nepalese rupees) per test for the *standard small panel *and U.S.$43 (5715 Nepalese rupees) per test for the *high-risk expanded panel*, because of our relatively small (825) sample size. Furthermore, we demonstrated that other perceived challenges were of diminished importance because we were able to demonstrate that scalable logistics have been established for the collection, transport, and analysis of samples with an acceptable turnaround time. Concerns of inadequate collection and processing can be overcome with appropriate staff training. In fact, even in countries where universal NBS is established, staff training is an integral part of the onboarding process. The ability to contact families was not a major obstacle; all families were contacted with positive and negative results because the standardized form attached to each NBS test includes current parent contact information.

There are valid concerns regarding the unavailability of comprehensive management, and in an LMIC it is not uncommon to find projects vying for limited resources, anxiety associated with the diversion of funds from other areas of the healthcare system, and governmental regulatory/policy agencies constrained by the reality of competing priorities of pediatric/neonatal care. Despite this, we think that it is still possible to establish a government-led universal NBS in Nepal. Other low-middle-income countries, for example, India and the Philippines, have successfully implemented universal NBS in public hospitals where the cost of services, including NBS tests, are free [24, 25].

Although many individual IEMs are uncommon, together they represent a group of disorders that contribute to significant morbidity and mortality within a population. It is also important to consider the knock-on effect in diagnosing these conditions, since some causative factors, like IEMs and genetic anomalies, may recur in future pregnancies [26]; hence, NBS is a simple and effective screening tool for the selection of high-risk pregnancies that would benefit from additional surveillance during the antenatal/intrapartum period, enabling timely referral and access to facilities with superior obstetric and neonatal care, thus ensuring health promotion and disease mitigation by early newborn testing and management. Many cases of IEMs are likely misdiagnosed, with unexplained deaths being attributed to more commonly occurring causes like infection, respiratory/cardiovascular diseases, and sudden infant death. The possibility of IEMs is not considered in initial diagnosis because some of these disorders may mimic severe sepsis which is still an important cause of morbidity and mortality. Without a concerted effort to determine the prevalence and distribution of IEMs across race and ethnicity in Nepal, there will be no incentive to diagnose and treat these disorders that may imitate more common conditions, thus NMR may remain above the United Nation’s targeted goal. Defining the problem should be the first step in augmenting physician training of IEMs, in medical school/post-graduate training, to create the expertise needed in Nepal over time. In our study survey, physicians indicated that the conditions tested are not significant contributors to mortality, but this was because we limited our test panel to conditions that can be managed in Nepal, with a focus on establishing logistics that show NBS is feasible for early diagnosis and treatment. To determine the prevalence of IEMs it will likely be necessary to perform testing using the *high-risk expanded panel *for a limited period and subsequently customize the testing panel to the needs of the Nepali population. Alternatively, screening of children/adults with intellectual disability could yield information about previously unknown diagnoses for these patients and contribute to the growing knowledge of IEMs and their prevalence in the population. Ideally, a combination of both prospective high-risk expanded NBS panels, as well as retrospective screening of known patients with intellectual disability, may provide valuable information to pediatricians. Identifying the magnitude of the problem and distribution among various ethnic groups would promote the implementation of appropriate regional healthcare planning policies within Nepal.

One of the anticipated challenges for capturing the entire newborn population for NBS is that approximately 40% of deliveries occur at home despite the government’s monetary incentive for in-hospital deliveries. Involvement and training of home-birth midwives will be paramount for the success of a universal NBS program, and will ultimately strengthen the primary healthcare system nationwide. Another challenge revolves around cultural practices and the scope of health literacy education required for parents. We speculate that universal NBS will be a driver to modification of the healthcare infrastructure that supports the follow-up of families after detection of an IEM.

## Conclusions

Our study shows that NBS is both feasible and acceptable in Nepal. The introduction of NBS in Kathmandu has heightened physician awareness and may result in the organic growth of NBS testing. The relatively substantial cost of testing in our study should be placed in the context of the small sample size, therefore implementation of a universal NBS program would benefit from the advantage of economies of scale that would drive down unit-test pricing. A government-sponsored NBS program will be a key factor in establishing NBS in Nepal, because the lack of a government subsidy may exacerbate health disparities/inequities due to variations in clinical practices, structural healthcare factors, and social norms.

NBS in Nepal aligns with the WHO *Every Newborn Action Plan* [27] aimed at accelerating progress to end preventable neonatal death and stillbirths, by using strategies associated with effective cause-specific interventions, and has the potential to be an efficient screening tool towards achieving the UNSDG of reducing the NMR in Nepal. NBS could potentially inform the direction of targeted public health policies for governmental agencies and improve population health outcomes.

## Supplementary Information

**Additional file 1: **STROBE Statement—Checklist of items that should be included in reports of cohort studies.

## Data Availability

Descriptive data is provided as part of the submitted manuscript. The data that support the findings of this study are not openly available due to reasons of sensitivity and are available from the corresponding author upon reasonable request (j-khan@northwestern.edu). Data are located in controlled access data storage at Kathmandu Medical College Teaching Hospital, Kathmandu, Nepal.

## Abbreviations

NMR: Neonatal mortality rate
LMIC: Low- and middle-income countries
NBS: Newborn metabolic screening
IEM: Inborn error of metabolism
WHO: World Health Organization
CAH: Congenital adrenal hyperplasia
G6PD: Glucose-6-phosphate dehydrogenase deficiency
DBS: Dried blood spot
NICU: Neonatal intensive care unit
